# Executive functions in post-traumatic stress disorder: their relation to PTSD severity and daily functioning

**DOI:** 10.3389/fpsyt.2025.1620472

**Published:** 2025-11-17

**Authors:** Nehal Safi, Elias Jahjah, Eyal Bergmann, Eyal Fruchter, Yael Caspi, Udi Oren, Naomi Josman, Evelyne Klinger, Batya Engel-Yeger

**Affiliations:** 1 Department of Occupational Therapy, Faculty of Social Welfare and Health Sciences, University of Haifa, Haifa, Israel; 2 Department of Psychiatry, Rambam Medical Center, Haifa, Israel; 3 Eye Movement Desensitization and Reprocessing (EMDR) Israel Association, Ra’anana, Israel; 4 Presence & Innovation Laboratory, Laval, France

**Keywords:** posttraumatic stress disorder (PTSD), behavioral regulation, executive functions, daily functioning, virtual shopping task performance, and severity of PTSD symptoms

## Abstract

**Background:**

Executive function difficulties may be prevalent in people with PTSD, and they might negatively affect their behavior and daily functioning. However, knowledge about the implications of executive function deficits on daily functioning and the assessments that reflect functional limitations is limited. This study aimed to measure how executive function difficulties affect daily functioning in PTSD using ecologically valid assessments that imitate daily activities.

**Methods:**

The participants were 66 individuals aged 18–65 years: 26 diagnosed with PTSD and 40 healthy controls. All participants completed a socio-demographic questionnaire, the Clinician-Administered PTSD Scale, and the Behavior Rating Inventory of Executive Functions—BRIEF-A, a self-reported tool to assess the impact of executive functions on daily activities. The participants also performed a virtual shopping activity to assess executive functions while performing in a daily activity using the Virtual Action Planning Supermarket (VAP-S 2), a novel, ecologically valid, performance-based tool.

**Results:**

Difficulties in executive functions were significantly more prevalent in PTSD patients: the BRIEF-A reflected how executive function difficulties manifested in behavioral dysregulation, restricting daily functioning. The VAP-S 2 revealed difficulties in executive functions, expressed as higher impulsivity, lower strategy use, and decreased performance efficiency while shopping in the virtual supermarket. Correlations were found between BRIEF-A and VAP-S 2: more collisions and lower inhibition in VAP-S 2 were correlated with worse BRIEF-A scores. Difficulties in executive functions correlated with PTSD severity.

**Conclusions:**

Executive function difficulties in PTSD may correlate with PTSD severity and restrict daily functioning. Therefore, executive functions in PTSD should be evaluated using ecologically valid assessments to illuminate the implications of real-life activities. VAP-S 2 uniquely enables an objective assessment of executive functions in real-life scenarios for PTSD, complementing self-report and enhancing the ecological validity of findings. It is recommended to combine self-reports that reflect the person’s authentic perspective with performance-based assessments, such as the VAP-S 2, to focus intervention on people’s real-life context and, by that, improve their function and well-being.

## 1 Introduction

Posttraumatic stress disorder (PTSD) may arise following a trauma resulting from experiencing, witnessing, or learning of actual or threatened death, serious injury, or sexual violence. The global PTSD prevalence is estimated at 4%–11%, with higher rates among veterans and high-risk professionals like emergency medical personnel and firefighters (1). PTSD is a chronic and progressive condition. The symptoms include re-experiencing, avoidance, and increased arousal, such as hypervigilance and irritability. As a result, PTSD interferes with daily activities, social and family relationships, work attendance, income, and lower educational and occupational success (1) and may significantly reduce people’s quality of life (QOL) (2, 3). Intervention efficiency is frequently limited, and the suffering that people with PTSD experience severely affects their life experience, inclusion in society, self-efficacy, and well-being (4, 5). Therefore, knowledge about PTSD symptoms and their implications on daily life should be elaborated to improve intervention success and enable people to experience better function and QOL. There is a need for new evaluation and intervention approaches that reflect the implications of PTSD symptoms on people’s daily lives (6, 7).

It is crucial to recognize the significant influence of executive functions (EF) on PTSD symptoms and their effects on daily life. EF difficulties in PTSD may contribute to the prevalent and dominant symptoms of PTSD—hyperarousability, attention deficits, and regulation difficulties (8–13). EF include planning, cognitive flexibility, problem-solving, inhibitory and emotional control, working memory, initiation, organization, self-monitoring, and task-monitoring (10, 14). Hence, EF allow the person to control goal-directed thoughts and behavior. EF are required to perform and participate in self-directed activities effectively, and they are essential for almost all activities in various daily situations and environments (15). In PTSD, the prevalent EF difficulties include, for example, difficulties in divided and selective attention, cognitive flexibility, inhibition, working memory, and planning (10, 14).

Studies also highlight that EF difficulties in PTSD correlate with PTSD severity (6) and enhance it—for example, impaired attention and inhibition contribute to hypervigilance and arousal symptoms (8, 16). EF difficulties in PTSD may also deteriorate emotional status (14), an individual’s healthcare maintenance, involvement in the intervention (14, 17, 18), and most importantly, reduced daily function and inclusion in society (2, 14, 17, 18).

According to the International Classification of Functioning, Disability, and Health (ICF) framework of the World Health Organization (19), the primary outcome measures of intervention efficiency are the person’s ability to perform daily activities and participate in life settings. However, evaluation and intervention for PTSD are mainly performed in clinical settings and focused on psychological and pharmacological treatment. Although intervention may reduce PTSD symptoms, in many cases, there is no complete remission (7, 20), and intervention does not fully address the implications on daily life functioning.

Based on the ICF and the significant negative impacts of EF difficulties on the daily function of people with PTSD (18, 21, 22), greater attention should be given to the role of EF in PTSD pathology and implications on daily function. Nonetheless, studies on EF difficulties data about EF in PTSD have been mainly gathered by neuropsychological evaluations with a focus on specific EF components, such as working memory, inhibition (for example, go/no-go) (18, 23), as measured in laboratory settings. Studies with ecologically valid assessments that reflect implications on daily life are scarce (24–26). Several studies on the daily activities of people with PTSD include self-report tools like the Canadian Occupational Performance Measure (COPM), Short Form (SF-36), and Activity Card Sort (ACS) (2, 25, 27). Self-report tools can increase the awareness of performance and challenges (28). However, they may not fully capture real-life task performance or daily functional impairments and may be influenced by self-awareness, mood, or bias. Applying ecological evaluation to assess the implications of EF in daily life may facilitate a deeper understanding of the daily challenges faced by PTSD patients, support the development of personalized interventions to enhance daily functioning in real-life settings, and elevate overall well-being.

One effective ecological platform for treating PTSD is virtual reality (VR), which is considered a highly motivated, safe, and readily available environment (29–32). Previous studies that employed VR, whether as an exposure therapy or evaluation tool, have primarily demonstrated VR’s utility in assessing and diagnosing PTSD symptoms (33, 34). Applying VR to PTSD assessment can be a convenient experience, reducing the sense of threat and optimizing emotional engagement for treatment with fewer side effects (33, 35, 36). However, studies utilizing VR to address everyday activities in PTSD, such as shopping or driving, as an objective and ecological tool are limited. Existing studies primarily focus on the relationship between performance and symptoms (24, 36, 37). Research lacks information about objective and ecological assessments that reflect how PTSD characteristics, such as difficulties in EF, affect daily activity performance.

The Virtual Action Planning Supermarket (VAP-S 2) (38) aims to answer this need and bridges the gap between laboratory assessment and daily life demands. VAP-S 2 offers an innovative, virtual, ecologically valid VR platform that simulates everyday activities, such as shopping, providing objective, performance-based data that have not previously been explored in PTSD populations. By combining BRIEF-A and VAP-S 2, this study captures both the perceived and actual executive function difficulties, thereby enhancing the ecological validity and clinical relevance of findings in PTSD.

The VAP-S 2 is used in rehabilitation to assess instrumental activities of daily living (IADL). It provides a comprehensive record of temporal and spatial aspects that reflect performance, while depending on EF, and therefore elucidates how EF support and interfere with activity performance (39, 40). Studies have shown its effectiveness in assessing performance differences in conditions like Parkinson’s disease, stroke, mild cognitive impairment, and schizophrenia (38, 41–43). These studies described the potential advantages of the VAP-S for rehabilitating people with deficits in EF (40). Nevertheless, studies utilizing the VAP-S 2 in PTSD are scarce.

This study aimed to elaborate on the knowledge about EF difficulties in people with PTSD and their implications on daily functioning. The specific aims are as follows: (1) to compare EF between people with PTSD and healthy controls using ecological evaluation tools: a self-report about EF in daily scenarios and the actual performance-based virtual assessment—the VAP-S 2 shopping task; among people with PTSD, (2) to examine the correlations between EF as measured in a self-report and the actual performance-based daily activities (shopping in a supermarket) using the virtual reality platform; and (3) to examine the relationship between EF and PTSD severity.

The study hypotheses are as follows: (1) EF difficulties would be significantly more prevalent in people with PTSD; among people with PTSD, (2) greater difficulties in EF would significantly correlate with lower efficiency while performing a shopping activity in the virtual supermarket; and (3) difficulties in EF would significantly correlate with symptom severity.

## 2 Method

### 2.1 Participants

This study included 66 participants aged 18–65 years: 26 participants with a diagnosis of PTSD who were outpatients in the Psychiatry Unit and 40 healthy participants recruited by snowball sampling and advertisements published in the same geographic area as the study group. The exclusion criteria (based on the medical records) were physical/psychiatric diagnosis that is not PTSD, severe health condition (such as cancer, cerebrovascular accident (CVA)), cognitive decline diagnosis, use of drugs/substances in the study group (except the use of prescription drugs for PTSD), and ADHD symptoms in the control group (a T-score higher than 65 in the CAARS*™*) (44). Because of the high prevalence of ADHD in people with PTSD, ADHD was not an exclusion criterion in the PTSD group (6, 45, 46). However, it was considered a covariate in the data analysis.

**Table 1** presents the participants’ socio-demographic data. No significant differences were found between the groups regarding marital status, co-residence, and socio-demographic status (**Table 1**). However, there was a significant uneven gender distribution, with a higher prevalence of men in the study group. There were also differences between groups in age (the mean age of the control group is nearly a decade lower than that of the study group), in education (70% of the controls have an academic degree, compared to 15% among the study group), and in employment (only 27% of the study group participants are employed, compared to 95% of controls). It is worth noting that since the study group comprised chronic PTSD outpatients, the differences in education and employment are important as PTSD can impact participation in these areas (3, 47). Variances regarding age, gender, and education were considered in the statistical analysis process.

**Table 1:** Socio-demographic characteristics (frequencies and differences) of the study groups.

Characteristic		(*n* = 66)	PTSD, (*n* = 26)	Control, (*n* = 40)	t
Mean (SD)					
Age		38.48 (12.13)	44.27 (10.75)	34.73 (11.59)	*t* = 3.362***
		(*n* = 66)	PTSD, (*n* = 26)	control (*n* = 40)	*χ* ^2^
*n* (%)					
Gender					9.179**
	Male	44 (67%)	23 (88%)	21 (52%)	
	Female	22 (33%)	3 (12%)	19 (48%)	
Marital status					1.531
	Single	23 (35%)	7 (27%)	16 (40%)	
	Married	37 (56%)	17 (65%)	20 (50%)	
	Widowed	0 (0%)	0 (0%)	0 (0%)	
	Divorced	6 (9.1%)	2 (7.7%)	4 (10%)	
Co-residence					.201
	Family	47 (71%)	18 (69%)	29 (72%)	
	Partner	9 (14%)	4 (15%)	5 (12%)	
	Parents	7 (11%)	3 (12%)	4 (10%)	
	Alone	3 (4.5%)	1 (3.8%)	2 (5.0%)	
	Other	0 (0%)	0 (0%)	0 (0%)	
Sociodemographic status					1.958
	Low	19 (29%)	10 (38%)	9 (22%)	
	Average + high	47 (71%)	16 (62%)	31 (78%)	
Education level					24.420***
	Incomplete secondary education (12 years or less)	18 (27%)	15 (58%)	3 (7.5%)	
	High school graduate (“Bagrut” diploma)	11 (17%)	5 (19%)	6 (15%)	
	Professional diploma	5 (7.6%)	2 (7.7%)	3 (7.5%)	
	Academic	32 (48%)	4 (15%)	28 (70%)	
Years of education					17.921***
	≤12	25 (37.8%)	18 (69.23%)	7 (17.5%)	
	>12	41 (62.1%)	8 (30.7%)	33 (82%)	
Currently employed		46 (70%)	9 (35%)	37 (92%)	24.999***

**p ≤.01; ***p ≤.001.

### 2.2 Instruments

The sociodemographic health questionnaire included information about participants’ health status, sociodemographic status, medications, and treatments.

The Conner’s Adult ADHD Rating Scales (CAARS-S:S) (44) is a self-report questionnaire designed to evaluate and screen ADHD in everyday life. The CAARS-S:S includes 26 items. For each item, the respondents indicate whether the described behavior occurred “never = 0”, “once in a while = 1”, “often = 2”, or “very frequently = 3” recently. Based on the sum of the scale, norms by age and gender (adjusted T-scores) were calculated. Higher scores indicate greater symptoms of ADHD. In this research, the CAARS-S:S was used to screen for symptoms of ADHD (when found, ADHD was held as a covariate). The cutoff for the abnormal ADHD performance range is higher than 65. Differences in ADHD symptoms between groups were taken into consideration in the statistical analysis.

The Clinician-Administered PTSD Scale CAPS (48) is the gold standard in PTSD assessment. The CAPS-5 is a 30-item structured interview that can be used to assess the DSM-5 PTSD symptoms. The questions cover the onset and duration of symptoms, subjective distress, impact on functioning, improvement since the last assessment, overall PTSD severity, and specifications for the dissociative subtype. A specific traumatic event must be identified for symptom inquiry, and standardized questions are used for each symptom. The full interview takes 45–60 min. In this study, the CAPS was administered by a trained psychiatric professional.

The Behavior Rating Inventory of Executive Function–Adult Version (BRIEF-A) (49) is a self-report questionnaire that was designed to assess EF in everyday life. The BRIEF-A includes 75 items. For each item, the respondents indicate whether the described behavior occurred “never = 1”, “sometimes = 2”, or “often = 3” in the past month. It includes nine clinical scales (inhibition, shift, emotional control, self-monitoring, initiation, working memory, plan, task-monitoring, organization) divided into two indices (the behavior regulation index (BRI) and metacognition index (MI)), which together make up the global executive composite (GEC) score. Adjusted T-scores (norms by age) are calculated based on the sum of each subscale and the general subscales. Higher scores indicate greater difficulties in EF. The abnormal EF performance range cutoff is a GEC score of 65 and above (49). The questionnaire was translated into Hebrew and adapted to fit the cultural context (50).

The Virtual Action Planning Supermarket (VAP-S 2) (38) is an upgraded version of the VAPS, with new graphic software that enables an adjustable environment designed to assess EF while shopping in a supermarket. The VAP-S 2 simulates a fully textured, medium-sized supermarket with multiple aisles. The task is to purchase seven items from a clearly marked products list displayed on the computer screen while facing multiple distractions commonly present in a daily environment, such as a supermarket (for example, a virtual buyer and randomized background noises) (51, 52). Then, the person has to proceed to the cashier’s desk and pay for the purchased products. A training task, similar but not identical to the test, is also available to enable users to become acquainted with the virtual environment. The VAP-S 2 yields 21 outcome measures: i.e., time (s) (initialization time, time to collect, checkout time, time to pay, exit time, stops time, session time), distance (in meters) (distance covered, distance in collecting, number of stops, collisions), and incorrect actions including checkout errors, other errors, perseverations, view the cart, need assistance, and intrusions (52). In this study, the outcome measures were conceptualized in terms of executive functioning into two categories, namely: (1) activity performance (mission completion)—measured by the number of purchased products and correct actions and (2) efficiency—the ability to complete the task with minimum expenditure of time and effort, measured by time, distance, and incorrect actions (25, 39). Additional outcome measures included impulsivity—a higher score indicated a higher level of impulsivity, strategy use (range 0–6)—a higher score indicated a greater ability to use strategies, and categorization (0/1/2 scores; categorizing the products that share a similar quality, e.g., banana and tomato are fruits)—a higher score indicates better categorization ability. Studies found that VAP-S 2 significantly differed between clinical populations and healthy adults (39, 40, 42). The VAP-S 2 displays culturally adjusted grocery items. The VAP-S 2 records the session and displays the task trajectory in upper or first-person view, providing an additional perspective of the track used during the activity.

### 2.3 Procedure

Ethical approval was obtained from the Helsinki Committee at Rambam Medical Center and the Institutional Review Board of the Faculty of Social Welfare and Health Sciences, University of Haifa. Data collection began on September 20, 2020 and continued until June 27, 2022. Individuals who were interested in participating in this study contacted the research coordinator. After approving the inclusion criteria, one or two meetings (in cases of fatigue impact) were scheduled in a quiet room at the psychiatric department (for the PTSD group) or at the participant’s home (for the control group). The participants signed a consent form in this meeting, completed the clinical questionnaires and the BRIEF-A, and performed the VAP-S 2.

### 2.4 Data analysis

Data were analyzed using SPSS (version 27). Descriptive statistics were performed to characterize the demographic data and the outcome measures. Tests of normality found a normal distribution in BRIEF-A scores, whereas the VAP-S 2 measures were not normally distributed. We used the MANCOVA test for the comparison hypotheses regarding the BRIEF-A while controlling for age, gender, and the number of education years as covariates. The effect size was determined according to Becker (53) and Richardson (53, 54). For the VAP-S 2 measures, we used the Mann–Whitney test. The correlations were examined using Pearson’s and Spearman’s correlation tests while controlling for the ADHD index and the symptoms’ severity variances. Due to multiple comparisons, the significance level was adjusted accordingly, and the tests were corrected for all pairwise comparisons using the Bonferroni correction. This resulted in a corrected significance threshold of *p* < 0.005 for the BRIEF-A analysis (MANCOVA test) and *p* < 0.004 for the VAP-S 2 analysis (Mann–Whitney test). This correction controls the family-wise error rate, reducing the risk of false positives that can occur when conducting multiple statistical tests.

## 3 Results

### 3.1 Participants’ clinical profile in the PTSD group

Based on the CAPS measure, 84.6% of the participants reported severe PTSD symptoms. Additionally, 87.5% reported severe symptoms in cluster B (intrusive thoughts and re-experiencing), 95.8% in cluster C (avoidance), 91.7% in cluster D (negative alteration in cognitions and mood), and 66.66% in cluster E (arousability). The most prevalent trauma type in the study group was war-related PTSD—including cases among veterans and combatants—accounting for 46% of the participants (**Table 2a**).

**Table 2a:** Descriptive statistics, means, and standard deviations of the characteristics of the PTSD group.

	PTSD			
		Range		
	n	Minimum	Maximum	Mean (SD)
Time since the trauma	26	1	48	15.92 (12.04)
How many years has been recognized in the therapeutic system?	26	1	23	5.88 (5.03)
			n (%)	
Type of the treatment—research evaluation				
	Medication		6 (23.1)	
	Medication + psychotherapy		9 (34.6)	
	Medication + OT therapy		5 (19.2)	
	Medication + psychotherapy + OT therapy		6 (23.1)	
Type of the trauma	Accident (transportational, natural)		5 (19.2)	
	Physical assault		4 (15.4)	
	Sexual assault		2 (7.7)	
	War-related PTSD		12 (46.2)	
	Occupational trauma (firefighters, police officers, paramedics, medical staff)		3 (11.5)	
	*Range*			
	n	Minimum	Maximum	Mean (SD)
Clinician-Administered PTSD Scale (CAPS) total for PTSD	24	13	65	50.16 (11.9)
Cluster B^2^	24	.60	3.60	2.7 (.78)
Cluster C^2^	24	1.00	4.00	2.9167 (.73)
Cluster D^2^	24	.29	3.80	2.5690 (.74)
Cluster E^2^	24	.00	3.00	2.1944 (.71)
		Severe, n (%)	Not severe, n (%)	
Severity of PTSD?		22 (84.61)	2 (7.69)	
Cluster B severity	24	21 (87.5)	3 (12.5)	
Cluster C severity	24	23 (95.8)	1 (4.2)	
Cluster D severity	24	22 (91.7)	2 (8.3)	
Cluster E severity	24	20 (66.66)	4 (33.33)	

Scores in CAPS Scale cluster (B, C, D, E) ≥2 signify severe PTSD symptoms.

Cluster B, re-experiencing; cluster C, avoidance; cluster D, negative alteration in cognitions and mood; cluster E, arousability.

Conner’s ADHD index indicated a significant difference between the groups, with a higher prevalence of ADHD symptoms in the PTSD group (about 1.5 times higher than among the control group). The participants in the control group had normal range scores in Conner’s ADHD index test (**Table 2b**).

**Table 2b:** Descriptive statistics, means, and standard deviations of the Conner`s ADHD index between the groups.

	PTSD				Control				*t*
	Range				Range				
	n	Minimum	Maximum	Mean (SD)	n	Minimum	Maximum	Mean (SD)	
ADHD index^2^	25	50	87	73.32 (9.54)	40	31	59	44.5 (7.729)	13.349***

T-score in ADHD index >65 indicates that the individual might have ADHD symptoms. ***p ≤. 001.

### 3.2 Differences in executive functions between groups based on BRIEF-A

Differences between groups were examined by the MANCOVA test controlling for age, gender, and the number of education years as covariates. The significance level was set at *p* ≤. 005 to account for multiple comparisons (**Table 3**).

**Table 3:** Differences between groups in executive functions according to BRIEF-A.

BRIEF subscale	(*n* = 66)	PTSD (*n* = 26)	Control (*n* = 40)	F (1, 61)	*η*_p_²
Mean (SD) [Range: minimum–maximum]					
Inhibition	63.59, (11.10)	73.77 (9.67), [56–91]	56.98 (5.60), [48–69]	42.864***	.413
Shift	65.41, (7.30)	70.19 (6.23), [57–79]	62.30 (6.23), [51–75]	7.372	.108
Emotional control	62.18, (10.72)	72.96 (6.39), [61–82]	55.17 (6.12), [43–69]	43.331***	.415
Self-monitoring	57.30, (11.80)	66.23 (12.16), [46–87]	51.50 (7.05), [42–68]	8.081	.117
Initiation	58.58, (17.89)	76.15 (12.31), [50–94]	47.15 (9.83), [37–78]	55.851***	.478
Working memory	59.89, (18.49)	78.19 (15.44), [43–97]	48.00 (6.98), [39–66]	60.788***	.499
Plan	63.71, (12.70)	76.12 (9.81), [58–93]	55.65 (6.17), [45–71]	52.810***	.464
Task monitoring	58.76, (16.20)	73.92 (14.13), [38–95]	48.90 (7.50), [36–66]	39.948***	.396
Organization	57.44, (12.44)	68.12 (11.55), [43–83]	50.50 (6.85), [42–69]	30.152***	.331
Behavior Regulation Index (BRI)	65.18, (11.52)	75.73 (7.81), [62–89]	58.33 (7.72), [49–91]	25.703***	.296
Metacognition Index (MI)	61.20, (16.31)	77.08 (12.99), [50–97]	50.88 (7.60), [40–75]	57.276***	.484
General executive composite (GEC)	63.86, (14.98)	79.31 (10.32), [59–96]	53.83 (6.58), [45–76]	63.463***	.510

Group differences were analyzed using the MANCOVA test while controlling for age, gender, and years of education as covariates. The significance level was adjusted to *p* ≤.005 to account for multiple comparisons. ***p ≤.001.

The prevalence of EF difficulties based on the GEC cutoff score was significantly higher among the PTSD group (T-score ≥65), 88.5%, compared to the control group, 5%. When examining the differences between groups in BRIEF-A scales, lower EF abilities were found in the PTSD group in most scales (**Table 3**). Additional predictive analyses are provided in the **Supplementary Material**.

### 3.3 Differences between groups in EF based on VAP-S 2

Differences between groups were examined by the Mann–Whitney test (**Table 4**). The significance level was set at *p* ≤. 004 to account for multiple comparisons.

**Table 4:** Executive function differences between groups based on their performance in the Virtual Action Planning Supermarket (VAP-S 2).

	Outcome measure	(*n* = 66)	PTSD (*n* = 26)	Control (*n* = 40)	*Z*
Mean (SD), Med (IQR = interquartile range), [range: minimum–maximum]					
Time (seconds)	Initialization time	3.21 (3.61), 2.20 (1.29),	5.05 (5.25), 4.20 (3.15),	2.02 (.59), 2.01 (.69),	-4.829***
		[.72–28.69]	[1.70–28.69]	[.72–3.38]	
	Time to collect	536.86 (333.32), 412.94 (1,473.77),	755.82 (410.41), 671.13 (707.65),	394.54 (156.40), 338.95 (178.38),	-4.016***
		[224.29–1,698.06]	[258.90–1,698.06]	[224.29–955.25]	
	Checkout time	49.74 (14.80), 44.88 (78.26),	52.75 (17.91), 46.80 (11.37),	47.78 (12.22), 43.79 (5.58),	-2.100
		[19.05–97.31]	[19.05–97.31]	[36.40–96.84]	
	Time to pay	6.82 (14.54), 3.88 (3.78)	12.07 (22.32), 5.92 (8.34),	3.40 (1.65), 3.32 (2.48),	-3.346***
		[.00–115.27]	[.00–115.27]	[1.09–8.25]	
	Exit time	18.85 9.50), 17.72 (11.91),	17.47 (10.79), 17.21 (12.10),	19.74 (8.58), 18.39 (10.67),	-.735
		[.00–57.91]	[.00–42.92]	[8.50–57.91]	
	Stops time	223.54 (170.70), 155.93 (140.27),	331.40 (215.83), 258.56 (419.34),	153.44 (76.86), 137.91 (79.11),	-3.714***
		[42.50–883.69]	[97.82–883.69]	[42.50–439.90]	
	Session Time	615.47 (350.04), 479.68 (1,537.51),	843.15 (429.34), 753.40 (747.81),	467.47 (170.35), 409.58 (182.35),	-3.950***
		[288.27–1,825.78]	[318.04–1825.78]	[288.27–1,072.47]	
Distance (meters)	Distance covered	202.54 (111.92), 158.45 (633.57),	282.85 (146.80), 230.54 (184.83),	169.85 (44.76), 146.43 (52.35),	-4.081***
		[111.70–745.27]	[125.88–758.04]	[121.68–299.55]	
	Distance in collecting	214.37 (112.19), 172.29 (106.32)	271.14 (146.42), 245.53 (180.02),	157.95 (44.25), 157.49 (53.05),	-4.068***
		[121.68–758.04]	[119.47–745.27]	[111.70–84.55]	
	Number of stops	15.39 (10.46), 12.00 (10.00)	21.92 (12.82), 18.50 (22.50),	11.15 (5.50), 10.00 (5.00),	-3.564***
		[2.00–47.00]	[7.00–47.00]	[2.00–30.00]	
	Collisions	5.45 (8.33), 2.00 (6.25),	10.92 (10.99), 7.00 (12.25),	1.90 (2.31), 1.00 (3.00),	-4.474***
		[.00–40.00]	[.00–40.00]	[.00–9.00]	
Mission completion	Correct actions	12.61 (1.41), 13.00 (.00)	12.00 (2.14), 13.00 (1.25),	13.00, 13.00 (.00),	-3.704***
		[4–13]	[4.00–13.00]	[13.00]	
	Correct purchases	6.74 (.95), 7.00 (0)	6.35 (1.44), 7.00 (1.00),	7.00 (.00), 7.00 (.00),	-3.438***
		[1–7]	[1.00–7.00]	[7.00]	
	Mission complete	19.35 (2.34), 20.00 (0),	18.35 (3.54), 20.00 (2.00),	20.00 (.00), 20.00 (.00),	-3.705***
		[5–20]	[5.00–20.00]	[20.00]	
Incorrect actions	Incorrect actions	8.21 (11.14), 4.00 (8.75),	14.00 (15.22), 5.50 (21.25),	4.45 (4.64), 3.00 (6.75),	-2.245
		[.00–58.00]	[.00–58.00]	[.00–19.00]	
	Checkout errors	.68 (2.95), .00 (.00),	1.27 (4.50), .00 (1.00),	.30 (1.07), 00 (.00),	-1.494
		[.00–23.00]	[.00–23.00]	[.00–6.00]	
	Other errors	.00 (.00), .00 (.00),	.00 (.00), .00 (.00),	.00 (.00), .00 (.00),	.000
		[.00]	[.00]	[.00]	
	Perseverations	4.18 (6.99), 1.00 (4.25)	7.73 (9.54), 3.00 (15.50),	1.87 (3.03), .50 (2.00),	-2.733
		[.00–32.00]	[.00–32.00]	[.00–14.00]	
	View the cart	.15 (.44), .00 (.00),	.27 (.60), .00 (.00),	.08 (.27), .00 (.00),	-1.483
		[.00–2.00]	[.00–2.00]	[.00–1.00]	
	Need assistance	.50 (1.03), .00 (1.00),	.88 (1.42), .00 (1.25),	.25 (.54), .00 (.00),	-1.9135
		[.00–5.00]	[.00–5.00]	[.00–2.00]	
	Intrusions	1.05 (1.31), 1.00 (2.00),	1.54 (1.68), 1.00 (2.00),	.72 (.88), .50 (.00),	-2.074
		[.00–7.00]	[.00–7.00]	[.00–3.00]	
	Impulsivity	8.70 (11.72), 5.00 (8.75)	15.23 (15.81), 6.50 (22.25),	4.45 (4.64), 3.00 (1.00),	-2.929**
		[.00–63.00]	[.00–63.00]	[.00–19.00]	
	Strategies use	3.55 (1.62), 4.00 (3.00),	2.15 (1.52), 2.00 (2.25)	4.45 (.88), 5.00 (1.00),	-5.445***
		[.00–6.00]	[.00–5.00]	[2.00–6.00]	
	Efficiency^4^	838.05 (457.33), 665.67 (509.09),	1,140.00 (564.50), 1,042.84 (898.33),	641.78 (209.81), 563.14 (214.92),	-4.108***
		[424.54–2,425.47]	[455.69–2,425.47]	[424.54–1,375.02]	

Differences between groups, frequencies, and percentages of the variables according to VAP-S 2.					
		(*n* = 66)	PTSD (*n* = 26)	Control (*n* = 40)	*χ* ^2^
		Prevalence (%)			
Exit					6.551**
Incorrect		4 (6.1%)	4 (15.4%)	0	
Correct		62 (93.9%)	22 (84.6%)	40 (100%)	
Categorization					14.173***
No categorization		3 (4.5%)	3 (11.5%)	0	
One categorization		16 (24.2%)	11 (42.3%)	5 (12.5%)	
Two categorizations		47 (71.2%)	12 (46.2%)	35 (87.5%)	

**p ≤.01; ***p ≤.001. ^4^a higher score reflects poorer efficiency.

Differences between groups were analyzed using the Mann-Whitney test. To account for multiple comparisons, the significance level was established at p ≤ 0.004.

When examining EF during VAP-S 2 performance, the participants with PTSD showed higher impulsivity, low strategy, and categorization use. They also showed interruptions in maintaining the sequence and continuity of the performance with higher mean scores in intrusions (sensory and visual interruptions), preservations (repeated actions), check-out errors, and longer exit time, although these differences were not statistically significant. All of these outcomes result in a longer time to complete the activity, longer trajectory distance, and more incorrect actions, leading to a significantly lower efficiency in completing the task (**Table 4**).

**Figure 1:**
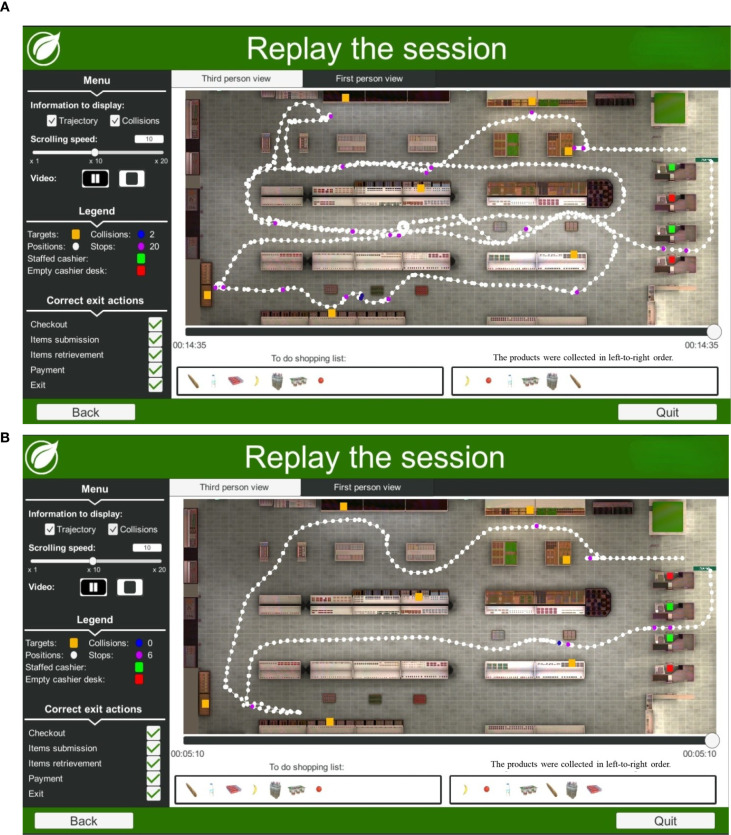
**(A)** Example trajectory of a 32-year-old man with PTSD performing the Virtual Action Planning Supermarket (VAP-S 2). This participant had an incomplete secondary education (12 or fewer education years) and completed the task in 14:35 min. An examination of the purchased items from the shopping list suggests that the participant categorized the groceries into dairy and fruit products and missed one product. **(B)** Example trajectory of a 33-year-old healthy man performing the Virtual Action Planning Supermarket (VAP-S 2). This participant had an incomplete secondary education (12 or fewer education years) and completed the task in 05:10 min. An examination of the purchased items from the shopping list suggests that the participant categorized the groceries into dairy and fruit products and collected all of the products.

**Figure 2:**
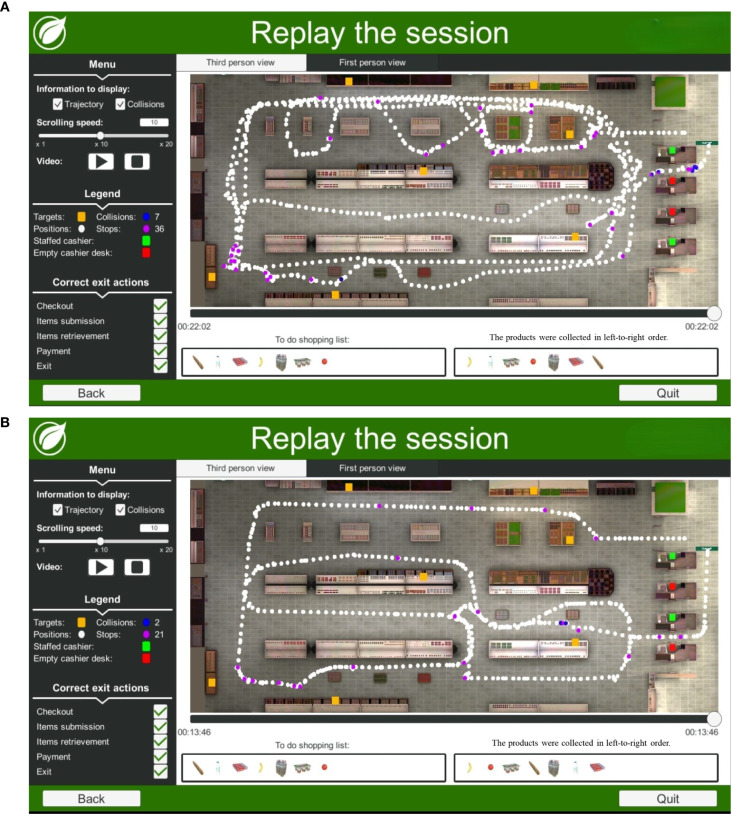
**(A)** Example trajectory of a 52-year-old man with PTSD while performing the Virtual Action Planning Supermarket (VAP-S 2). This participant had a professional diploma (12 to 13 years of education) and completed the task in 22:02 min. An examination of the purchased items from the shopping list suggests that the participant categorized the groceries into dairy products only and collected all of the products. **(B)** Example trajectory of a 52-year-old healthy man performing the Virtual Action Planning Supermarket (VAP-S 2). This participant had a high school graduate (“Bagrut” diploma) (12 education years). The total time to complete the task was 13:46 min. An examination of the purchased items from the shopping list suggests that the participant categorized the groceries into fruit products only and collected all of the products.

**Figure 3:**
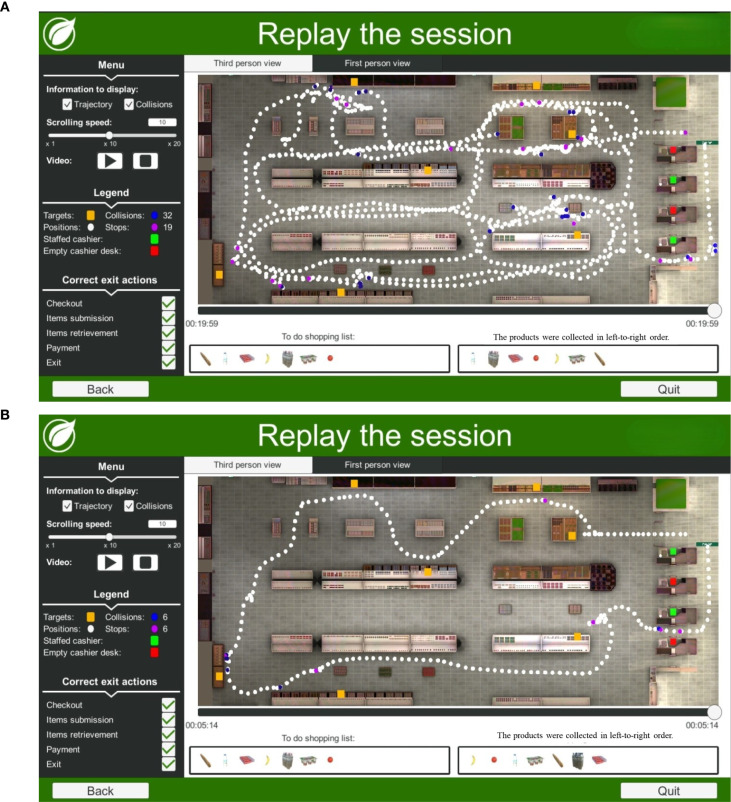
**(A)** Example trajectory of a 39-year-old man with PTSD while performing the Virtual Action Planning Supermarket (VAP-S 2). This participant had an incomplete secondary education (12 years or less) and completed the task in 19:59 min. An examination of the purchased items from the shopping list suggests that the participant categorized the groceries into fruit products only and collected all of the products. **(B)** Example trajectory of a 37-year-old healthy man performing the Virtual Action Planning Supermarket (VAP-S 2). This participant had an academic education (15 years of education or more) and completed the task in 05:14 min. An examination of the purchased items from the shopping list suggests that the participant categorized the groceries into dairy and fruit products and collected all of the products.

**Figures 1**–**3** present differences in performance in VAP-S 2 between PTSD patients and healthy participants. As shown in **Figure 1A**, the trajectory of a 32-year-old man with PTSD performing the VAP-S 2 indicates less efficient performance with longer time to complete the activity, longer trajectory distance, and more incorrect actions compared to **Figure 1B**, the trajectory of a 33-year-old healthy man performing the VAP-S 2.

In **Figure 2A**, the trajectory of a 52-year-old man with PTSD performing the VAP-S 2 task indicates less efficient performance characterized by a longer time to complete the activity, greater trajectory distance, and more incorrect actions compared to **Figure 2B**, which shows the trajectory of a 52-year-old healthy man performing the VAP-S 2.

In **Figure 3A**, the trajectory of a 39-year-old man with PTSD performing the VAP-S 2 task indicates less efficient performance, characterized by a longer time to complete the activity, greater trajectory distance, and more incorrect actions compared to **Figure 1B**, the trajectory of a 37-year-old healthy man performing the VAP-S 2.

### 3.4 Correlations between EF as measured by the self-report (BRIEF-A) and actual performance-based assessment (VAPS 2) among the study group

Controlling for PTSD severity and ADHD, significant correlations were found between BRIEF-A scores and VAP-S 2 performance. Higher BRIEF-A general executive composite (GEC) and emotional control scores correlated with more collisions in the VAP-S 2 shopping task [*r*_s_(19) = 0.478, *p* < 0.05; *r*_s_(19) = 0.548, *p* < 0.01]. Poorer self-monitoring and organization correlated with more errors at checkout and exit selection [*r*_s_(19) = 0.446, *p* < 0.05; *r*_s_(19) = 0.457, *p* < 0.05]. Worse planning related to increased intrusions, reflecting difficulty ignoring distractions during task performance [*r*_s_(19) = 0.436, *p* < 0.05]. Additionally, lower inhibition and organization scores were associated with a greater need for assistance to perform [*r*_s_(19) = -0.6, *p* < 0.01; *r*_s_(19) = ;-0.435, *p* < 0.05]. Note that higher BRIEF-A scores indicate worse performance.

### 3.5 Correlations between EF and PTSD severity among the study group

Controlling for ADHD, PTSD severity significantly correlated with lower EF on the BRIEF-A—initiation, task-monitoring, and metacognition index (MI). Specifically, arousability (cluster E) significantly correlated with lower initiation, working memory, task-monitoring, MI, and GEC. In addition, negative alteration in cognition and mood (cluster D) significantly correlated with lower initiation (**Table 5**).

**Table 5:** Correlations between EF and PTSD severity in the PTSD group.

BRIEF-A subscale	Total CAPS PTSD severity ^a^	Cluster B severity ^a^	Cluster C severity ^a^	Cluster D severity ^a^	Cluster E severity ^a^
Inhibition	.057	.180	-.228	.094	.083
Shift	.139	-.063	.363	.207	.152
Emotional control	.033	.109	.200	-.089	.110
Self-monitoring	.005	.184	.217	-.111	.044
Initiation	.461*	.168	.131	.470*	.619**
Working memory	.381	.268	.194	.250	.477*
Plan	.322	.263	.306	.225	.345
Task-monitoring	.554**	.408	.285	.389	.575**
Organization	.107	.299	.039	-.088	.099
Behavior Regulation Index (BRI)	.057	.155	.169	.003	.111
Metacognition Index (MI)	.434*	.320	.217	.378	.460*
General executive composite (GEC)	.383	.337	.252	.241	.456*

*p ≤.05; **p ≤.01.

Correlations between the BRIEF-A and the PTSD severity according to the CAPS while controlling for Conner`s ADHD index.

Scores in CAPS Scale cluster (B, C, D, E) ≥2 signify severe PTSD symptoms.

a Cluster B, re-experiencing; cluster C, avoidance; cluster D, negative alteration in cognitions and mood; cluster E, arousability.

## 4 Discussion

This study focused on EF difficulties in people with PTSD, their relation to PTSD severity, and their implications on daily functioning. The study also highlighted the relevance of combining self-reports and performance-based ecologically valid evaluations to reflect EF difficulties in daily life scenarios. This significant information may optimize intervention efficiency.

The study’s first aim was to compare EF between participants with PTSD and healthy controls using the BRIEF-A self-report and an actual performance-based evaluation via a VR platform—the first hypothesis was confirmed. A higher prevalence of EF difficulties was found in the PTSD group in both measures—in most BRIEF-A scales as well as the behavioral regulation scale. The virtual shopping task highlighted that EF difficulties were expressed in higher impulsivity and lower strategy use, which affected performance efficiency. Previous reports exist regarding EF difficulties in PTSD and note, for example, how impulsivity impacts the ability to stop and monitor and how reduced inhibitory control affects the ability to deal with internal and external distractions (10, 11, 18) as manifested in the present study results of the VAPS 2 when distractions such as a virtual buyer and randomized noises appear while performing the VR shopping task. Impaired ability to manage distractions in PTSD can be associated with hyperactivity in the salience network (SN)—a circuit that detects and prioritizes significant internal and external stimuli. In PTSD, the SN (including the amygdala, anterior insula, and dorsal anterior cingulate cortex) shows heightened activation, lowering the threshold for perceiving stimuli as salient. This leads to exaggerated responses to distractions and increased sensitivity to environmental cues. Such neural alterations underlie core symptoms of hypervigilance and may contribute to the executive dysfunction observed in PTSD (55–58). The BRIEF-A used in the present study also provided a detailed profile of the various EF components affected in PTSD. This is important since many previous studies have used assessments that measure a specific EF component (for example, Trail Making Test B (TMT B) or Digit Span total (DS-tot)) (14, 59, 60), lacking a general picture that reflects daily life.

The present study used both a self-report and an actual performance of a daily activity to illuminate the functional implications of EF deficits in people with PTSD. This is one of the first studies to apply the VAP-S 2. Both BRIEF-A and VAP-S 2 were found to be feasible for profiling EF difficulties in people with PTSD. The few previous studies that used the BRIEF-A with people with PTSD (61) also found BRIEF-A to be a sensitive assessment to manifest EF difficulties and recommended using it in intervention and rehabilitation. The present study highlights the relevance of adding an assessment of the actual performance of a daily activity, such as shopping in the supermarket via the VAP-S 2.

The results of the VAP-S 2 confirmed that people with PTSD might have lower performance efficiency than healthy controls. Studies that used the VAP-S 2 in PTSD are scarce. This emphasizes the relevance of the present study and the VAP-S 2 in elaborating ways to evaluate how EF difficulties in people with PTSD impact their daily function and activity performance. The results showed that even a frequent and common activity, such as shopping in a supermarket, may be challenging for people with PTSD (22, 62, 63), placing significant demands on their executive functions. VAP-S 2 succeeded in depicting how, in this instrumental ADL activity, various EF components such as initiation, planning, attention, selection, working memory, sequencing, and monitoring are impaired in PTSD. This might explain their lower efficiency, lower ability to complete the task, higher impulsivity, incorrect actions, and lower use of strategies while performing the VAP-S 2. However, it is important to acknowledge that ADHD comorbidity may influence EF outcomes (6, 45, 46), and further research is needed to explore how EF affects daily activity performance in PTSD.

With regard to the second hypothesis, the VAP-S 2 also correlated with the BRIEF-A scores, showing concurrent validity—for example, a higher number of collisions (i.e., participants bumping into objects/people while performing the virtual shopping task) correlated with greater difficulties in EF (GEC score) and lower emotional control; lower self-monitoring and organization abilities were related to a higher number of errors in VAP-S 2. Lower inhibition and organization correlated with a greater need for assistance when performing the shopping task. Hence, using both a self-report, such as BRIEF-A, and the actual activity performance via VAP-S 2 may reflect how difficulties in EF are expressed in real-life scenarios that require proper planning, strategy use, rule compliance, problem-solving, and precise regulation of distractive stimuli (39–42). Furthermore, these findings remain significant after controlling for the ADHD covariate, strengthening our results and aligning with prior research highlighting the overlapping, yet distinct, EF impairments such as inhibition seen in PTSD and ADHD (6, 46).

Interestingly, in the present study, BRIEF-A scores correlated with PTSD severity (mainly arousability). As opposed to BRIEF-A, none of the VAP-S 2 measures correlated with PTSD symptom severity. While some previous studies indicate that PTSD symptoms may worsen EF (12, 64) and daily activity performance (23, 65), others claim that EF difficulties in PTSD are not moderated by PTSD symptom severity. Once PTSD occurs, EF difficulties are presented and not necessarily related to PTSD severity (9, 18, 60). In our study, it may be suggested that VAP-S 2 requires integral complex cognitive abilities that do not necessarily correlate with PTSD severity but with PTSD existence. Furthermore, most of the study participants were war-related PTSD including veterans and combatants (46% of the participants) and men. This may contribute to the current results as a previous study found that poorer EF performance in PTSD was related to male gender, older patients, war trauma, and comorbidity with depression that restricted participation and inclusion into society (14). Further studies on men and women in larger samples should examine the relation between VAP-S 2 outcomes and its relation with BRIEF-A and PTSD severity.

Studies should also explore the potential advantage of evaluating EF while performing a daily life activity, such as shopping in VAP-S 2, which offers promising clinical advantages for PTSD care, particularly by enabling the evaluation and training of activities that require executive function (EF) within realistic, daily-life contexts, such as shopping. The VAP-S 2 seems to have several benefits, especially for people with PTSD: it is a user-friendly environment that can be customized to the patient and the intervention plan. It may decrease the patient’s sense of threat and optimize emotional engagement in treatment (33, 36). Through the practice of familiar, everyday tasks via VAP-S 2, individuals with PTSD can build confidence, increase functional independence, and strengthen the courage to participate actively in their communities. These advances can help reduce stigmatization and foster social inclusion (32–34). This aligns with the International Classification of Functioning Disability and Health of the World Health Organization (19), which includes measures that imitate daily life activities that may improve intervention outcomes in terms of better independence and participation in daily life activities. As VAP-S 2 and similar VR platforms become increasingly accessible and affordable, their integration into routine clinical practice creates new opportunities for personalized interventions, ongoing progress monitoring, and improved real-world functional outcomes.

## 5 Conclusion

EF difficulties are more prevalent in people with PTSD and may affect their daily functioning. These impairments may also correlate with the severity of the symptoms, which may also impede daily functioning. The Virtual Action Planning Supermarket (VAP-S 2) uniquely enhances EF assessment by offering an immersive virtual reality environment that mimics real-life tasks, such as shopping. This method complements traditional self-report measures, increasing ecological validity and sensitivity to functional impairments relevant to patients’ daily activities. EF evaluation should combine self-reports that provide an authentic perspective based on the person’s own voice with actual performance-based assessments that imitate activities in real-life settings. Furthermore, incorporating VAP-S 2 in clinical settings can improve the evaluation of EF, make better tailor rehabilitation efforts, and provide a secure environment for practicing daily life skills. This combined approach provides a thorough understanding of perceived and actual executive function challenges, guiding personalized clinical interventions and enhancing treatment outcomes.

## 6 Limitations

Given the challenges of recruiting and retaining participants with PTSD, this study involved a relatively small sample size, which may have limited the ability to detect some “true effects” or associations between PTSD and EF. The sample had a gender imbalance, with more men included, since the inclusion criteria and professional considerations led to the exclusion of several women with PTSD. This imbalance may limit the generalizability of the findings to broader populations. Additionally, unmeasured or uncontrolled variables may have influenced the findings, potentially masking relationships between PTSD symptoms and executive function outcomes. The cross-sectional nature of the study, with variability in participant characteristics—such as trauma type, which was not analyzed as a primary variable due to heterogeneity and sample size constraints—further limits the generalizability of the results. Future research with larger, more demographically balanced samples is necessary to strengthen these findings and enhance their applicability.

## Data Availability

The raw data supporting the conclusions of this article will be made available by the authors, without undue reservation.
