# Supplementary material for: Executive functions in post-traumatic stress disorder: their relation to PTSD severity and daily functioning
Source: Front Psychiatry. 2025 Nov 17;16:1620472. doi: 10.3389/fpsyt.2025.1620472 (PMC12667252; doi:10.3389/fpsyt.2025.1620472)
Supplement: Supplementary file 1 [file Table1.pdf]

Executive Functions in Post-Traumatic Stress Disorder: Their Relation to PTSD Severity and Daily Functioning

Safi N, Jahjah E, Bergmann E, Fruchter E, Caspi Y, Oren U, Josman N, Klinger E and Engel-Yeger B (2025) Executive functions in post-traumatic stress disorder: their relation to PTSD severity and daily functioning. Front. Psychiatry 16:1620472.
doi: 10.3389/fpsyt.2025.1620472

Supplementary Material for the paper:

Supplementary Analysis Using MANCOVA with Covariates:

MANCOVA test was conducted as the data analysis to examine group differences while controlling for covariates that were statistically different between groups, specifically age, gender, and years of education. Recognizing that substantial differences in covariates can introduce bias, we performed additional predictive analyses to evaluate the potential impact of these covariates on the outcomes (Miller & Chapman, 2001).

The results indicate that **almost all of the dependent** variables (BRIEF-A in this case) were not predicted by the covariates, suggesting that the covariates did not account for the observed group differences. However, caution is still advised due to the initial imbalance in covariates, these findings support the appropriateness of including MANCOVA in the data analysis process.

As seen in Table 1, emotional control significantly predicted age, accounting for 16.2% of the variance. Self-monitoring significantly predicted gender accounting for 15.8% of the variances. Two variables significantly predicted years of education. Emotional control accounted for 25.7% of the variances and shift contributed an additional 4.8%.

Table 1 - Prediction of BRIEF-A by age, gender, and education years.

	Model 1			Model 2		
Variable	**B**	**SE B**	**β**	**B**	**SE B**	**β**
**Age**						
Emotional control	0.474	0.128	0.418***			
R^2		0.162				
F(1,64) for change in R^2		13.581***				
**Gender**						
Self monitoring	-0.017	0.005	-0.413***			
R^2		0.158				
F(1,64) for change in R^2		13.190**				
**Years of education**						
Emotional control	-0.024	0.005	-0.518***	-0.15	0.006	0.337**
Shift				-0.2	0.009	-0.3*
R^2		0.257			0.305	
F(1,64) for change in R^2		23.496***			5.366*	

*p<.05, **p<.01, ***p<.001

Executive Functions in Post-Traumatic Stress Disorder: Their Relation to PTSD Severity and Daily Functioning

Safi N, Jahjah E, Bergmann E, Fruchter E, Caspi Y, Oren U, Josman N, Klinger E and Engel-Yeger B (2025) Executive functions in post-traumatic stress disorder: their relation to PTSD severity and daily functioning. Front. Psychiatry 16:1620472.
doi: 10.3389/fpsyt.2025.1620472

Supplementary Analysis Using Mann-Whitney with Covariates:

Given the non-normal distribution of the dependent variable, a Mann-Whitney U test was conducted to compare the two groups. As significant demographic differences were observed, we acknowledge the limitations of this test in controlling for covariates. To address this, we conducted additional analyses using case matching to control for age, gender, and education years, which confirmed the robustness of our findings.

When we compared the differences between the matched subjects the results indicate that **almost all of the VAP-S 2 variables** were significantly different as seen in Table 2.

Table 4. Executive functions differences between groups based on their performance in the Virtual Action Planning Supermarket (VAP-S 2).

	Outcome measure	(N =66)[1,2,3]	PTSD, (N =26) [1,2,3]	Control, (N=40) [1,2,3]	Z
Time (seconds)	**Initialization time**	3.21(3.61), 2.20(1.29),	5.05(5.25), 4.20(3.15),	2.02(.59), 2.01 (.69),	-***
		[.72-28.69]	[1.70 - 28.69]	[.72 -3.38]	
	**Time to collect**	536.86 (333.32), 412.94 (1473.77),	755.82(410.41), 671.13(707.65)	394.54(156.40), 338.95(178.38),	-4.016***
		[224.29-1698.06]	[258.90 -1698.06]	[224.29 -955.25]	
	**Checkout time**	49.74 (14.80), 44.88 (78.26),	52.75(17.91), 46.80(11.37),	47.78(12.22), 43.79(5.58),	-2.100
		[19.05- 97.31]	[19.05 -97.31]	[36.40 -96.84]	
	**Time to pay**	6.82(14.54), 3.88 (3.78)	12.07(22.32), 5.92(8.34),	3.40(1.65), 3.32(2.48),	-3.346***
		[.00 -115.27]	[.00 -115.27]	[1.09 -8.25]	
	**Exit time**	18.85 9.50), 17.72(11.91)	17.47(10.79), 17.21(12.10),	19.74(8.58), 18.39(10.67),	-.735
		[.00 -57.91]	[.00- 42.92]	[8.50 -57.91]	
	**Stops time**	223.54(170.70), 155.93(140.27),	331.40(215.83), 258.56(419.34),	153.44(76.86), 137.91(79.11),	-3.714***
		[42.50 -883.69]	[97.82 -883.69]	[42.50 -439.90]	
	**Session Time**	615.47 (350.04), 479.68 (1537.51)	843.15(429.34), 753.40 (747.81),	467.47(170.35), 409.58 (182.35),	-3.950***
		[288.27 -1825.78]	[318.04- 1825.78]	[288.27 -1072.47]	
Distance (meters)	**Distance covered**	202.54(111.92), 158.45(633.57),	282.85(146.80), 230.54(184.83),	169.85(44.76), 146.43(52.35),	-4.081***
		[111.70 - 745.27]	[125.88 -758.04]	[121.68 -299.55]	
	**Distance in collecting**	214.37(112.19), 172.29(106.32)	271.14(146.42), 245.53(180.02),	157.95(44.25), 157.49(53.05),	-4.068***
		[121.68 - 758.04]	[119.47 -745.27]	[111.70 -84.55]	
	**Number of stops**	15.39(10.46), 12.00(10.00)	21.92(12.82), 18.50(22.50),	11.15(5.50), 10.00(5.00),	-3.564***

Executive Functions in Post-Traumatic Stress Disorder: Their Relation to PTSD Severity and Daily Functioning

Safi N, Jahjah E, Bergmann E, Fruchter E, Caspi Y, Oren U, Josman N, Klinger E and Engel-Yeger B (2025) Executive functions in post-traumatic stress disorder: their relation to PTSD severity and daily functioning. Front. Psychiatry 16:1620472.
doi: 10.3389/fpsyt.2025.1620472

Table 4. Executive functions differences between groups based on their performance in the Virtual Action Planning Supermarket (VAP-S 2).

	Outcome measure	(N =66)[1,2,3]	PTSD, (N =26) [1,2,3]	Control, (N=40) [1,2,3]	Z
		[2.00 -47.00]	[7.00 -47.00]	[2.00 -30.00]	
	**Collisions**	5.45(8.33), 2.00(6.25),	10.92(10.99), 7.00(12.25),	1.90(2.31), 1.00(3.00),	-4.474***
		[.00 -40.00]	[.00 -40.00]	[.00 -9.00]	
**Mission completion**	**Correct actions**	12.61(1.41), 13.00 (.00)	12.00(2.14), 13.00(1.25),	13.00, 13.00(.00),	-3.704***
		[4 -13]	[4.00 -13.00]	[13.00]	
	**Correct purchases**	6.74(.95), 7.00(0)	6.35(1.44), 7.00(1.00),	7.00(.00), 7.00(.00),	-3.438***
		[1-7]	[1.00 -7.00]	[7.00]	
	**Mission complete**	19.35(2.34), 20.00 (0),	18.35(3.54), 20.00(2.00),	20.00(.00), 20.00(.00),	-3.705***
		[5-20]	[5.00 -20.00]	[20.00]	
**Incorrect actions**	**Incorrect actions**	8.21(11.14), 4.00(8.75),	14.00(15.22), 5.50(21.25),	4.45(4.64), 3.00(6.75),	-2.245
		[.00 -58.00]	[.00 -58.00]	[.00 -19.00]	
	**Checkout errors**	.68(2.95), .00(.00),	1.27(4.50), .00(1.00),	.30(1.07), 00(.00),	-1.494
		[.00 -23.00]	[.00 -23.00]	[.00 -6.00]	
	**Other errors**	.00(.00), .00(.00),	.00(.00), .00(.00),	.00(.00), .00(.00),	.000
		[.00]	[.00]	[.00]	
	**Perseverations**	4.18(6.99), 1.00(4.25)	7.73(9.54), 3.00(15.50),	1.87(3.03), .50(2.00),	-2.733
		[.00 -32.00]	[.00 -32.00]	[.00 -14.00]	
	**View the cart**	.15(.44), .00(.00),	.27(.60), .00(.00),	.08(.27), .00(.00),	-1.483
		[.00 -2.00]	[.00 -2.00]	[.00 -1.00]	
	**Need assistance**	.50(1.03), .00 (1.00),	.88(1.42), .00(1.25),	.25(.54), .00(.00),	-1.9135
		[.00 -5.00]	[.00 -5.00]	[.00 -2.00]	
	**Intrusions**	1.05(1.31), 1.00 (2.00),	1.54(1.68), 1.00(2.00),	.72(.88), .50(.00),	-2.074
		[.00 -7.00]	[.00 -7.00]	[.00 -3.00]	
	**Impulsivity**	8.70(11.72), 5.00 (8.75)	15.23(15.81), 6.50(22.25),	4.45(4.64), 3.00(1.00),	-2.929**
		[.00 -63.00]	[.00 -63.00]	[.00 -19.00]	
	**Strategies use**	3.55(1.62), 4.00 (3.00),	2.15(1.52), 2.00(2.25)	4.45(.88), 5.00(1.00),	-5.445***
		[.00 -6.00]	[.00 -5.00]	[2.00 -6.00]	
	**Efficiency[4]**	838.05(457.33), 665.67(509.09),	1140.00(564.50), 1042.84 (898.33),	641.78(209.81), 563.14(214.92),	-4.108***
		[424.54 -2425.47]	[455.69 -2425.47]	[424.54 -1375.02]	

Differences between groups, frequencies, and percentages of the variables according to VAP-S 2.

	(N =66) Prevalence (%)	PTSD, (N =26) Prevalence (%)	Control, (N=40) Prevalence (%)	χ^2

Executive Functions in Post-Traumatic Stress Disorder: Their Relation to PTSD Severity and Daily Functioning

Safi N, Jahjah E, Bergmann E, Fruchter E, Caspi Y, Oren U, Josman N, Klinger E and Engel-Yeger B (2025) Executive functions in post-traumatic stress disorder: their relation to PTSD severity and daily functioning. Front. Psychiatry 16:1620472.
doi: 10.3389/fpsyt.2025.1620472

Table 4. Executive functions differences between groups based on their performance in the Virtual Action Planning Supermarket (VAP-S 2).

Outcome measure	(N =66)[1,2,3]	PTSD, (N =26) [1,2,3]	Control, (N=40) [1,2,3]	Z
**Exit**				6.551**
**Incorrect**	4(6.1%)	4(15.4%)	0	
**Correct**	62(93.9%)	22(84.6%)	40(100%)	
**Categorization**				14.173***
**No categorization**	3(4.5%)	3(11.5%)	0	
**One categorization**	16(24.2%)	11(42.3%)	5(12.5%)	
**Two categorizations**	47(71.2%)	12(46.2%)	35(87.5%)	

[1]Mean (SD), [2]MED (IQR= Interquartile Range), [3]Range [Minimum – Maximum]; **$p \leq .01$, ***$p \leq .001$. [4] a higher score reflects poorer efficiency.
